# 

**Published:** 1900-07

**Authors:** 


### International Dental Journal

JAMES TRUMAN, D.D.S., Editor.

GEORGE W. WARREN, D.D.S., Assistant Editor.

LIBRARY
Indexed
S. G. O.

VOL. XXI. JULY, 1900. No. 7.

PUBLISHED MONTHLY BY

THE INTERNATIONAL DENTAL PUBLICATION COMPANY,

624 CHESTNUT STREET, PHILADELPHIA.

EDITORIAL OFFICE, 4505 CHESTER AVENUE, PHILADELPHIA, PA.

$2.50 a Year, in Advance. Single Copies, 25 Cents.

Printed by J. B. Lippincott Company, Philadelphia.

Entered at the Post-Office at Philadelphia, and admitted for transmission through the mails at second-class rates.

